# Ageism Against Older Adults and Associated Factors: A Multicenter Cross‐Sectional Study Among Dental Students in Midwestern Brazil

**DOI:** 10.1111/eje.70153

**Published:** 2026-04-05

**Authors:** Eunice Ellen Gontijo de Menezes, Leonardo Marchini, Oscar Rysavy, Patrick Barlow, Cláudio Rodrigues Leles, Túlio Eduardo Nogueira

**Affiliations:** ^1^ Postgraduate Program in Dentistry, Faculty of Dentistry Federal University of Goiás (UFG) Goiânia Goiás Brazil; ^2^ Department of Preventive and Community Dentistry University of Iowa, College of Dentistry and Dental Clinics Iowa City USA; ^3^ Division of Biostatistics and Computational Biology University of Iowa, College of Dentistry and Dental Clinics Iowa City Iowa USA; ^4^ Department of Internal Medicine University of Iowa, Carver College of Medicine Iowa City Iowa USA

**Keywords:** aged, ageism, dental education, dental students, geriatric dentistry

## Abstract

**Objective:**

This study aimed to assess the occurrence of ageism towards older adults among dental students and investigate associated factors.

**Material and Methods:**

This cross‐sectional observational study included dental students who had completed at least one clinical academic component. Participants completed, in person, a printed self‐administered questionnaire consisting of the Brazilian version of the Ageism Scale for Dental Students (ASDS‐Braz), the Jefferson Scale of Empathy (JSE‐Br) and the Duke University Religion Index (DUREL), as well as items addressing sociodemographic characteristics, intergenerational aspects, and academic experiences. Bivariate and multivariable models were used to explore associations between independent variables and the ASDS‐Braz score. All analyses were performed in R version 4.4.1, adopting a 5% significance level.

**Results:**

A total of 795 dental students from seven Higher Education Institutions in Goiás, Brazil, participated in the study. Most participants were female (74.3%; *n* = 587), with a median age of 22.0 years (Q1 = 21.0; Q3 = 25.0). Only 35.6% reported previous exposure to geriatric dentistry content or related activities. Significant differences in ageism scores were observed between students with and without prior exposure to geriatric dentistry (*p* < 0.01), with those who had prior exposure demonstrating lower ageism scores. Additionally, a significant negative association was found between ageism and empathy scores (*p* < 0.001; β = −0.10), with higher empathy associated with lower ageism.

**Conclusions:**

Dental students who had previous academic exposure to geriatric dentistry or care for older adults demonstrated significantly lower ageism scores. Furthermore, higher levels of empathy were associated with fewer ageist attitudes, underscoring the need to integrate geriatric content and empathy development into dental education.

## 1 Introduction

The reduction in fertility and mortality rates, along with increased life expectancy, has contributed to a global demographic transition [1]. This shift is associated with advances in science, improved nutrition, urbanization, expanded access to healthcare services and the development of vaccines [1, 2]. As a result, health services are experiencing a significant increase in the number of geriatric patients.

Older adults often face multiple chronic conditions, polypharmacy, and cognitive decline, which can complicate the delivery of appropriate healthcare [3]. Additionally, age‐related prejudice—known as ageism—can negatively impact both the experiences and quality of care provided to this population. Coined in 1969 by Robert Butler [4], the term ‘ageism’ was later defined by the World Health Organization (WHO) in 2021 as ‘stereotypes (how we think), prejudice (how we feel), and discrimination (how we act) against people based on their age’ [5]. In the same year, the WHO published the Global Report on Ageism [5], which inspired a global campaign and action plan within the framework of the Decade of Healthy Aging (2021–2030) [6].

There is growing evidence that ageism also affects dental care, influencing both the delivery of care to older adults and how it is received [7, 8]. Previous studies suggest that ageist attitudes are linked to dentists' academic training. For instance, Swiss dental students in more advanced years of study tend to express fewer ageist attitudes [9]. Similarly, intergenerational contact appears to be a protective factor; a Greek study found that frequent interaction with older adults was associated with reduced ageist attitudes [10]. Sex has also been identified as a relevant factor, with female U.S. dental students showing lower levels of ageism [11].

To assess ageism in the dental context, researchers have developed a specific tool: the Ageism Scale for Dental Students (ASDS) [11]. This scale has undergone cross‐cultural validation and adaptation in several countries, including Greece [12], Romania [13], France [14], Serbia [15], Switzerland [16], Iran [17], India [18], Poland [19], Malaysia [20], Japan [21] and Brazil [22]. In Brazil, the ASDS was translated and culturally adapted with a sample of 156 students from the Southern region, with the Brazilian version (ASDS‐Braz) demonstrating acceptable validity and reliability [22].

Despite these efforts, findings on factors associated with ageism among dental students remain inconsistent. Moreover, many studies have employed general ageism scales that are not specific to the dental field. Even among studies that have used the ASDS, most have primarily focused on its validation and cross‐cultural adaptation, rather than on explanatory analyses. Although a few studies have examined variables such as empathy and religiosity, these factors remain relatively underexplored despite their potential relevance.

Previous research has shown that religiosity may play a relevant role in shaping attitudes towards older adults. Beyond its well‐documented association with health and well‐being, religiosity has been described as a potential contextual and individual‐level protective factor in the face of ageism. Cross‐national evidence suggests that more religious environments may buffer the negative effects of ageism on well‐being, possibly through the promotion of social support, prosocial norms, and values that emphasize respect for older individuals [23]. Moreover, a study of Greek dentists found that a stronger belief in God was associated with lower ageist attitudes, reinforcing the hypothesis that individual religiosity may be linked to more positive perceptions of older adults [10].

Brazil is characterized by significant sociocultural diversity across its regions, which may influence attitudes and behaviours towards older adults. To date, however, no studies on ageism among dental students have been conducted in the country's Midwest region, underscoring the need for regionally representative research. Understanding the problem and its associations is a pivotal step in devising strategies to combat ageism.

Therefore, this study aimed to assess the occurrence of ageism towards older adults and investigate associated factors among dental students in the metropolitan region of Goiânia, Goiás, Brazil. We hypothesized that ageism is present among dental students and may be associated with variables such as sex (with lower levels expected among female students), intergenerational contact (with frequent contact linked to lower ageism), religiosity and empathy (with higher levels associated with lower ageism), and prior exposure to geriatric dentistry content in their academic curriculum.

## 2 Material and Methods

### 2.1 Study Design and Sample

This was a cross‐sectional observational study conducted at dental schools in the state of Goiás, located in Midwestern Brazil. Data were collected between April and June 2023. The study is reported in accordance with the Strengthening the Reporting of Observational Studies in Epidemiology (STROBE) guidelines [24].

The research protocol was reviewed and approved by the Research Ethics Committee of the Federal University of Goiás (UFG), Brazil (approval number: 66109222.7.0000.5083). Additionally, formal authorization was obtained from all selected higher education institutions (HEIs) prior to data collection. All participants provided written informed consent.

The target population consisted of undergraduate dental students enrolled in institutions located in the metropolitan region of Goiânia, Goiás, Brazil. HEIs were selected from a list provided by the Regional Council of Dentistry of Goiás. Of the 29 identified dental programs, 22 were excluded due to reasons such as the absence of students in clinical disciplines, locations outside the metropolitan area of Goiânia, or failure to respond to the study invitation. Ultimately, seven HEIs were included—five located in Goiânia and two in neighbouring municipalities within the metropolitan region.

Inclusion criteria were as follows: being an undergraduate student enrolled in one of the selected dental programs, having completed at least one clinical discipline, and agreeing to participate in the study by signing the informed consent form. Foreign students without proficiency in Portuguese were excluded, as limited language skills could compromise their understanding of the study questionnaires.

A non‐probabilistic sampling method was used, in which all students present during visits to the selected HEIs who met the eligibility criteria were invited to participate. No formal sample size calculation was performed, and the final sample was treated as a census of eligible students.

### 2.2 Data Collection

Upon acceptance to participate in the study, each HEI's administration was asked to appoint a key informant to assist the researchers in planning the data collection process. This individual could be the program coordinator or another person designated by the coordination team.

During this preparatory phase, the key informant provided information such as the number of eligible students (based on the previously described criteria) and suggested strategic days and times for data collection. Data were collected in person, preferably in classroom settings; clinical outpatient settings were used only when no other feasible option was available for a given class.

Data were collected using printed, self‐administered questionnaires consisting of four sections: (1) the Brazilian version of the Ageism Scale for Dental Students (ASDS‐Braz) [22]; (2) the Brazilian student version of the Jefferson Scale of Medical Empathy (JSE‐Br) [25]; (3) items related to sociodemographic characteristics, academic background, and intergenerational interaction with older adults; and (4) the Brazilian version of the Duke University Religion Index (DUREL) [26, 27]. All questionnaires were distributed by the same researcher across all HEIs, following a standardized protocol developed by the research team. In each class, the researcher introduced herself, explained the study's objectives, and provided instructions on how to complete the questionnaires. Participants were informed that participation was voluntary and were encouraged to respond to all items.

In addition, program‐related information for each HEI was gathered. This step involved reviewing the institution's website to extract data such as program duration and the presence of mandatory and/or elective courses related to geriatric dentistry.

### 2.3 Pilot Study

Before data collection, a pilot study was conducted with 17 first‐year master's students enrolled in the Graduate Program in Dentistry at the Federal University of Goiás, Brazil. The average completion time was 20 min, and all questionnaires were returned fully completed.

The pilot study was used to test the application process, estimate average response time, and identify potential difficulties in understanding the questionnaires. All participants were asked to share their impressions of the questionnaire and were invited to provide written comments and suggestions. Based on their feedback, minor adjustments were made, primarily related to formatting.

### 2.4 Dependent Variable

Ageism was defined as the dependent variable (primary outcome) in this study. It was assessed using the Brazilian version of the Ageism Scale for Dental Students (ASDS‐Braz), originally developed by Rucker et al. (2018) [11] and cross‐culturally adapted to Brazil by Rucker et al. (2020) [22].

The ASDS‐Braz consists of 12 statements, including 3 reverse‐scored items, distributed across three domains: negative perception of older adults, positive perception of older adults, and complexity of elder care. Participants indicated their level of agreement with each item using a six‐point Likert scale: 1 = strongly disagree, 2 = disagree, 3 = slightly disagree, 4 = slightly agree, 5 = agree, and 6 = strongly agree. A total score was calculated by summing all item scores, with higher scores indicating a greater level of ageism [22].

### 2.5 Independent Variables

The independent variables included sociodemographic characteristics, academic background, intergenerational interaction with older adults, empathy and religiosity. Each of these variables is described below:

Sociodemographic data included age, sex, family monthly income, marital status, race/ethnicity and the primary place of residence during the participant's childhood. Academic background variables included the student's current semester in the undergraduate program and prior participation in activities related to Geriatric Dentistry or oral healthcare for older adults. Participants were also asked whether they were afraid of getting older and if they had heard of the term ‘ageism’.

Intergenerational interaction with older adults was assessed through a set of questions based on the participants' past experiences and living arrangements. These questions covered aspects such as the existence of older adults in the family, the quality of relationships with older adult family members, current or past cohabitation with older adults, previous close contact with older adults, frequency of contact, and caregiving experiences involving older adults.

Empathy was measured using the Brazilian student version of the Jefferson Scale of Medical Empathy (JSE‐Br) [25]. The scale consists of 20 items, including 10 reverse‐scored items, rated on a seven‐point Likert scale ranging from 1 (strongly disagree) to 7 (strongly agree). The total score is calculated by summing all item scores, yielding a range from 20 to 140, with higher scores indicating greater empathy [25]. Permission to use the scale was formally obtained from the copyright holders prior to conducting this study.

Religiosity was assessed using the Brazilian version of the Duke University Religion Index (DUREL) [26, 27], which evaluates three dimensions: organizational religious activity (ORA), non‐organizational religious activity (NORA), and intrinsic religiosity (IR), also referred to as subjective religiosity [26]. The instrument contains five items: one item each for ORA and NORA, and three items for IR. Responses are given on a five‐point Likert scale for IR and a six‐point scale for ORA and NORA. Scores range from 1 to 6 for ORA and NORA, and from 3 to 15 for IR, with higher scores indicating greater religiosity in each dimension [27].

### 2.6 Statistical Analyses

The ASDS‐Braz score was calculated by summing all item responses, with reverse scoring applied to negatively worded items. Non‐response to any single item resulted in an invalid total score for that participant. The JSE‐Br score was also computed by summing all items after reverse scoring where appropriate. All participants met the imputation criteria defined in the JSE‐Br scoring algorithm. The three dimensions of the DUREL were scored separately. Non‐response to any single item resulted in exclusion from the analysis of all DUREL domains. Internal consistency for each multi‐item scale was evaluated using Cronbach's alpha.

Descriptive statistics were calculated for all collected variables, including medians and interquartile ranges for continuous variables and proportions for categorical variables. Bivariate and multivariable models were used to explore associations between independent variables and the ASDS‐Braz score. Bivariate models were fit using all available data for each predictor.

To account for potential variability in scores across institutions, linear mixed‐effects models with a random intercept for school were employed in both bivariate and multivariable analyses. These models were fit using residual maximum likelihood (REML) estimation in the lme4 package (v1.1–35.5) [28]. Assumptions of normality and homoscedasticity were visually assessed using quantile‐quantile (Q–Q) plots and residuals versus fitted values plots. Coefficient significance was evaluated using *t*‐tests with Satterthwaite estimated degrees of freedom, implemented via the lmerTest package (v3.1–3) [29].

All analyses were performed in R, version 4.4.1 [30]. A significance level of 5% was adopted for all inferential tests.

## 3 Results

Of the seven participating HEIs, six were private institutions. Geriatric Dentistry was included in the curriculum only at the single public institution (Table S1). Table 1 presents the general characteristics of the study participants. The final sample comprised 795 dental students, enrolled in the 4th to 10th semesters of the Dentistry program, with a median age of 22.0 years (Q1 = 21.0; Q3 = 25.0). Most participants were female (*n* = 587; 74.3%) and single (*n* = 650; 82.4%). The majority self‐identified as white (*n* = 401; 50.9%) and reported having spent most of their childhood in urban areas, either in the state capital (*n* = 429; 54.3%) or in other towns (*n* = 327; 41.4%).

**TABLE 1:** General characteristics of the study participants (*n* = 795).

Variable	Categories	Values
Sex—*n* (%)	Female Male Missing	587 (74.3) 203 (25.7) 5
Age in years—median (Q1; Q3)	—	22.0 (21.0; 25.0)
Race/colour—*n* (%)	White Brown/mixed race Black Asian Indigenous Missing	401 (50.9) 300 (38.1) 46 (5.8) 40 (5.1) 1 (0.1) 7
Family monthly income (in Brazilian reais)—*n* (%)	Less than R$ 1000/month Between R$ 1000 and R$ 3000/month Between R$ 3001 and R$ 5000/month Between R$ 5001 and R$ 7000/month Between R$ 7001 and R$ 9000/month More than R$ 10 000/month Missing	15 (1.9) 77 (9.9) 217 (28.0) 69 (8.9) 157 (20.3) 239 (30.9) 21
Marital status—*n* (%)	Single Married In a civil union/domestic partnership Divorced Other Widowed Missing	650 (82.4) 94 (11.9) 24 (3.0) 14 (1.8) 6 (0.8) 1 (0.1) 6
Place of residence during the majority of childhood—*n* (%)	Urban area (capital) Urban area (outside capital) Rural area Other Missing	429 (54.3) 327 (41.4) 32 (4.1) 2 (0.3) 5
Current semester in the dental course—*n* (%)	4th semester 5th semester 6th semester 7th semester 8th semester 9th semester 10th semester Missing	31 (3.9) 211 (26.8) 80 (10.2) 252 (32.1) 43 (5.5) 161 (20.5) 8 (1.0) 9

Regarding prior involvement in activities related to Geriatric Dentistry or oral healthcare for older adults, only 282 participants (35.6%) reported having such experience, with participation in classes or courses lasting less than 10 h being the most frequently reported category (*n* = 137; 17.3%). Concerning intergenerational interaction with older adults, 96.3% of participants reported currently having older adults in their families, and the majority described their relationship as good or excellent (*n* = 712; 93.3%). Nearly half of the participants (*n* = 383; 49.6%) reported currently or previously living with older adults. When asked whether they had heard of ageism, 646 participants (84.1%) responded negatively. Further details are presented in Table 2.

**TABLE 2:** Participants' intergenerational interaction with older adults and prior academic experiences related to geriatric dentistry or oral healthcare (*n* = 795).

Variable	Categories	Values
Current existence of older adults in the family—*n* (%)	Yes No Missing	752 (96.3) 29 (3.7) 14
Evaluation of the relationship with older adults (for participants with older adults in the family)—*n* (%)	Great Good Indifferent Bad Terrible Not applicable Missing	553 (72.5) 159 (20.8) 15 (2.0) 6 (0.8) 1 (0.1) 29 (3.8) 32
Current or previous cohabitation with older adults—*n* (%)	Yes No Missing	383 (49.6) 389 (50.4) 23
Current or previous experience in caring for older adults—*n* (%)	Yes No Missing	393 (49.7) 397 (50.3) 5
Previous academic activity related to geriatric dentistry or oral health care for older adults—*n* (%)	Yes No Missing	282 (35.6) 511 (64.4) 2
Type of activity completed (for participants who answered yes to the variable above)—*n* (%)	Mandatory course in Geriatric Dentistry (yes) Elective course in Geriatric Dentistry (yes) Academic league or outreach project (yes) Geriatric dentistry congress (yes) Lecture, conference or course (< 10 h) (yes) Other type of activity (yes)	26 (3.3) 37 (4.7) 31 (3.9) 18 (2.3) 137 (17.3) 96 (12.1)
Previous experience providing care or assisting in the care of an older adult patient in a hospital bed/home or wheelchair?—*n* (%)	Yes No Missing	132 (17.6) 620 (82.4) 43
Previous close contact with older adults—*n*(%)	Yes No Missing	743 (94.2) 46 (5.8) 6
Has heard of ageism (age discrimination)—*n*(%)	Yes No Missing	122 (15.9) 646 (84.1) 27
Afraid of getting older—*n*(%)	Yes No Missing	257 (33.6) 508 (66.4) 30

Table 3 provides a descriptive summary of the scores on the empathy scale (JSE‐Br), the religiosity scale (DUREL), and the ageism scale (ASDS‐Braz). Additionally, Figure 1 provides a visual summary of the distribution of responses across all ASDS items, allowing identification of both the individual items and their respective response levels.

**TABLE 3:** Descriptive statistics of the ASDS‐BRAS, JSE‐Br, and DUREL scores.

Variable	Mean (SD)	Median (IQR)	Missing
Ageism—Total score of the Ageism Scale for Dental Students (ASDS‐BRAS)	37.8 (5.7)	38 (34, 42)	56
Empathy—Total score of the Jefferson Scale of Medical Empathy (JSE‐Br)	116.0 (12.7)	118 (109, 125)	0
Religiosity—Duke Religion Index (DUREL) score – ORA dimension Duke Religion Index (DUREL) score—NORA dimension Duke Religion Index (DUREL) score—IR dimension	3.2 (1.5) 3.2 (1.7) 3.7 (3.2)	3 (2, 4) 2 (2, 5) 3 (1, 4)	26

Abbreviations: IQR, Interquartile range; IR, intrinsic religiosity; NORA, non‐organizational religious activity; ORA, organizational religious activity; SD, Standard deviation.

**FIGURE 1:**
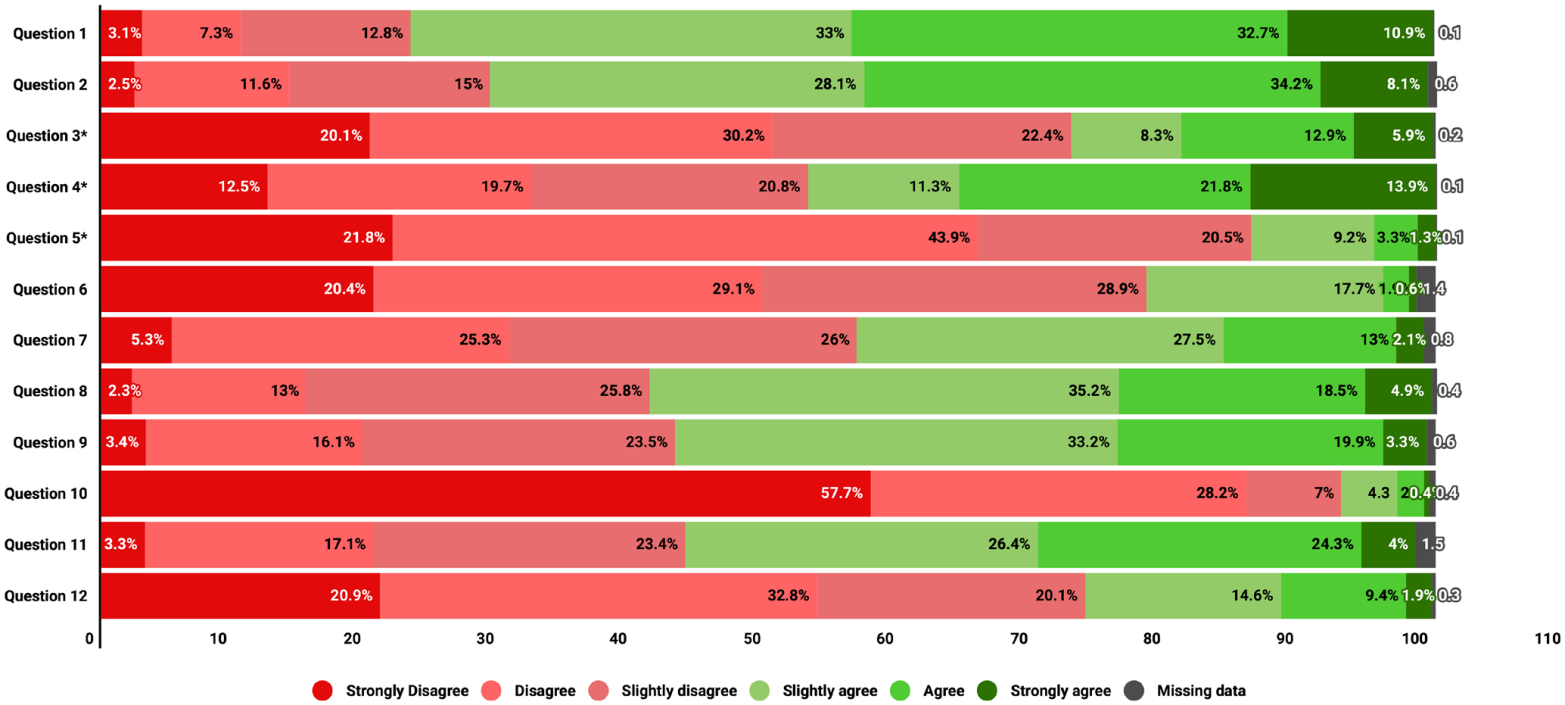
Distribution of participants' responses per item of the Ageism Scale for Dental Students (ASDS). Asterisks denote reverse‐scored items. The higher the proportion of red‐coloured cells, the greater the disagreement with ageist statements. Q1—Taking a medical history from elderly patients is frequently time‐consuming; Q2—Taking a medical history from elderly patients is frequently complex; Q3—I tend to pay more attention towards my elderly patients than my younger patients; Q4—I tend to have more sympathy towards my elderly patients than my younger patients; Q5—In general, elderly people contribute a lot to society; Q6—Elderly patients are better off in nursing homes; Q7—Elderly patients often will not accept recommended treatment plans; Q8—Elderly people do not take good care of their teeth; Q9—Elderly patients do not usually comply with dental advice; Q10—The elderly patient does not live long enough to make it worthwhile to invest money in expensive dental treatment; Q11—It is too costly to provide out of office dental care to homebound elderly patients; Q12—I tend to favour extractions over extensive restorative procedures when planning treatment for an elderly patient.

No association was observed between the total ageism score and any of the sociodemographic variables (Table 4). Participants who reported previous participation in activities related to Geriatric Dentistry or the care of older adults showed significantly lower ASDS‐Braz scores compared to those who had not (*p* = 0.002). Further details on intergenerational interaction and prior academic experience variables are presented in Table 5.

**TABLE 4:** Results of the bivariate analysis between ageism (ASDS‐Braz total score) and sociodemographic variables.

	Ageism total score (ASDS‐Braz)			
	Median (IQR)	Mean (SD)	*β* Coefficient (95% CI)	*p*
Sex				
Male	37 (34, 41)	37.4 (5.9)	—	—
Female	38 (34, 42)	38.0 (5.6)	0.53 (−0.39, 1.46)	0.258
Marital status				
Single	38 (34, 42)	37.8 (5.7)	—	—
Married	38 (33, 43)	38.0 (6.0)	−0.11 (−1.38, 1.17)	0.869
Other	38 (35, 41)	38.6 (5.5)	0.22 (−1.52, 1.95)	0.807
Race/colour/ethnicity				
White	38 (34, 42)	37.8 (5.7)	—	—
Brown/mixed race	37 (34, 41)	37.6 (5.9)	−0.27 (−1.14, 0.60)	0.540
Other	39 (34, 42)	38.6 (5.5)	0.42 (−0.95, 1.79)	0.551
Family monthly income (in Brazilian reais)				
Less than R$ 1.000 per month	37.5 (33, 43)	37.2 (6.2)	—	—
Between R$ 1.000 e R$ 3.000 per month	38 (33, 41)	37.3 (5.6)	0.45 (−2.78, 3.67)	0.786
Between R$ 3.001 e R$ 5.000 per month	38 (33, 42)	37.8 (5.8)	1.07 (−1.98, 4.12)	0.490
Between R$ 5.001 e R$ 7.000 per month	39 (33, 42.5)	38.5 (5.9)	1.68 (−1.57, 4.93)	0.310
Between R$ 7.001 e R$ 9.000 per month	38 (34, 41)	37.5 (6.4)	0.72 (−2.46, 3.80)	0.647
More than R$ 10.000 per month	37 (34, 41)	38.0 (5.2)	1.25 (−1.79, 4.29)	0.420
Place of residence during the majority of childhood				
Urban area (capital)	37 (33, 42)	37.6 (5.8)	—	—
Urban area (outside capital)	38.5 (34, 42)	38.0 (5.7)	0.11 (−0.74, 0.96)	0.801
Rural area/Other	40 (37, 42)	39.8 (5.5)	1.44 (−0.66, 3.53)	0.179
Age	—	—	−0.01 (−0.08, 0.06)	0.784

Abbreviations: CI, Confidence interval; IQR, Interquartile range; SD, Standard deviation.

**TABLE 5:** Results of the bivariate analysis of ageism (ASDS‐Braz total score) in relation to participants' intergenerational interaction with older adults and prior academic experiences in geriatric dentistry or oral healthcare.

	Ageism total score (ASDS)			
	Median (IQR)	Mean (SD)	*β* Coefficient (95% CI)	*p*
Current existence of older adults in the family				
No	37 (34, 42)	38 (6.2)	—	—
Yes	38 (34, 42)	37.9 (5.7)	0.01 (−2.32, 2.33)	0.996
Current or previous cohabitation with older adults				
No	38 (34, 41.5)	37.7 (5.6)	—	—
Yes	38 (34, 42)	37.9 (5.8)	0.30 (−0.52, 1.12)	0.474
Current or previous experience in caring older adults				
No	38.5 (34, 42)	38.0 (5.9)	—	—
Yes	38 (34, 41.5)	37.8 (5.5)	−0.19 (−1.00, 0.62)	0.645
Previous academic activity related to geriatric dentistry or oral health care for older adults				
No	39 (35, 42)	38.6 (5.7)	—	—
Yes	36 (33, 41)	36.6 (5.6)	−1.40 (−2.26, −0.53)	**0.002**
Has provided care or assisted care of an older patient				
No	38 (34, 42)	37.8 (5.8)	—	—
Yes	38 (33.5, 41)	37.5 (5.6)	0.06 (−1.03, 1.16)	0.909
Previous close contact with older adults				
No	38.5 (33.5, 43.5)	38.3 (6.4)	—	—
Yes	38 (34, 42)	37.8 (5.7)	−0.51 (−2.22, 1.20)	0.556
Has heard of ageism (age discrimination)				
No	38 (34, 42)	38.1 (5.5)	—	—
Yes	37 (32, 41)	36.6 (6.4)	−0.93 (−2.07, 0.22)	0.112
Afraid of getting older				
No	38 (33, 42)	37.6 (5.8)	—	—
Yes	39 (34, 42)	38.4 (5.6)	0.87 (0.01, 1.74)	0.049
Number of semesters attended			0.0006 (−0.28, 0.29)	0.997

Abbreviations: CI, Confidence interval; IQR, Interquartile range; SD, Standard deviation.

A one‐unit increase in a participant's empathy score was associated with an estimated 0.10‐unit decrease in the ageism score (*p* < 0.001). No significant associations were found between ageism and religiosity scores (Table 6).

**TABLE 6:** Results of the bivariate analysis considering the outcome ageism (ASDS‐Braz total score) and empathy (JSE‐Br) and religiosity (DUREL) measures.

	*β* Coefficient (95% CI)	*p*
JSE‐Br total score	−0.10 (−0.13, −0.07)	**< 0.001**
DUREL score—ORA dimension	−0.07 (−0.35, 0.21)	0.625
DUREL score—NORA dimension	0.11 (−0.14, 0.35)	0.400
DUREL score – IR dimension	−0.01 (−0.14, 0.12)	0.903

Abbreviations: CI, Confidence interval; IR, intrinsic religiosity; NORA, non‐organizational religious activity; ORA, organizational religious activity.

## 4 Discussion

This study assessed the occurrence of ageism towards older adults among dental students and explored potential associated factors. Our findings indicate a significant association between empathy and ageism, suggesting that students with higher empathy scores exhibited lower ageism scores. Additionally, students who reported prior participation in activities related to Geriatric Dentistry or the dental care of older adults showed significantly lower ageism scores than those without such experience.

These results are consistent with a study conducted in France involving 284 participants, including students and their families, which found that higher levels of empathy were significantly associated with reduced negative prejudice towards older adults [31]. A study conducted in Brazil with dental students also found a significant association between empathy and ageism, indicating that higher levels of empathy are associated with lower levels of ageism [32]. Empathy, recognized as a key element in building human connections and understanding others' experiences, plays a pivotal role in mitigating ageist attitudes [33]. In clinical settings, empathy contributes to the delivery of quality care for older patients, supporting thorough history taking and fostering a compassionate, responsive approach [34].

In our study, only one of the seven participating HEIs offered a formal Geriatric Dentistry component, and only 35.6% of students reported prior participation in an academic activity related to geriatric dentistry or oral healthcare for older adults. Furthermore, when asked about their prior knowledge of the term ageism, 84.1% of participants reported being unfamiliar with it. A lack of knowledge about aging and related issues has been strongly associated with more ageist attitudes, which is particularly concerning given the global trend of population aging and its implications for healthcare systems [35, 36]. Universities, which play a critical role in shaping future healthcare professionals, often fail to adequately address issues related to aging and ageism in their curricula [37, 38]. Our finding that students with prior exposure to geriatric content demonstrated lower levels of ageism underscores the importance of integrating such content into dental education to better prepare professionals for the care of an aging population.

No association was found between sex and ageism scores in this study, in contrast to previous research suggesting that female students are generally less ageist [10, 11]. A possible explanation for this discrepancy lies in the sample composition, which was predominantly female (over 73%), potentially limiting the variability necessary to detect differences by sex.

Religiosity has been linked to higher levels of empathy and positive attitudes towards older adults in several studies [39, 40, 41]. Some religious traditions have been empirically associated with more positive perceptions of older adults, particularly regarding their social and personal value, as higher levels of religiosity and specific religious practices have been linked to more positive attitudes towards older people [42]. A study of Greek dentists found that stronger belief in God was associated with lower ageist attitudes [10]. However, our study did not find an association between religiosity and ageism scores. This discrepancy may be partly explained by differences in study populations and measurement instruments, as the previous study evaluated practicing dentists and employed a different religiosity measure, limiting direct comparison.

Beyond identifying the occurrence and associated factors of ageism, it is essential to develop strategies to combat it. Although ageism has long been ingrained in society, the COVID‐19 pandemic brought renewed attention to this issue [43]. During this period, public policies aimed at addressing ageism were strengthened, particularly in response to extreme forms of ageism such as senicide, defined as the murder or abandonment to death of older adults [44]. Another front of the battle was the rationing of healthcare services for older individuals, a topic that gained prominence during the pandemic [45]. In dentistry, the shortage of specialists in Geriatric Dentistry and the limited presence of related coursework in undergraduate programs can also be viewed as expressions of structural ageism that must be addressed.

Multiple strategies have been proposed to combat ageism. Legislative action to protect older adults is one important approach. Additionally, educational interventions, particularly those that increase knowledge about aging, have proven effective in reducing ageist attitudes [35, 46]. Academic institutions have been identified as ideal settings for such interventions [47]. Intergenerational contact, especially in‐person interactions with older adults, has also demonstrated positive outcomes [47]. Burnes et al. [48] found that combining intergenerational contact with educational content on aging yields the strongest effects in reducing ageism.

While this study benefited from several methodological strengths, including a large sample size and the use of a standardized data collection using validated scales and questionnaires, it also presents limitations. The cross‐sectional design precludes causal inference, and the regional focus (Midwestern Brazil) limits generalizability. Given that ageism seems to be shaped by cultural and contextual factors, future research should explore the phenomenon in other regions of Brazil to broaden the evidence base. Additionally, intervention studies are needed to evaluate the effectiveness of specific educational and experiential strategies aimed at reducing ageism among dental students. Qualitative research is also recommended, as ageism is a cross‐cultural phenomenon. In‐depth studies could better explore how cultural background, religiosity, empathy and intergenerational experiences shape age‐related attitudes and help inform more comprehensive strategies to address ageism.

Our findings carry important implications for dental education and clinical practice. They highlight the urgent need to incorporate Geriatric Dentistry components into undergraduate curricula and to adopt more comprehensive educational approaches that promote not only technical proficiency but also empathy, ethical values and interpersonal skills.

## 5 Conclusions

In conclusion, this study identified that dental students who had previously participated in academic activities related to Geriatric Dentistry or the care of older adults exhibited significantly lower ageism scores. Additionally, higher levels of empathy were associated with a reduction in ageism scores.

## Author Contributions

Eunice Ellen Gontijo de Menezes: conceptualization, methodology, formal analysis, investigation, resources, writing – original draft, visualization. Leonardo Marchini: conceptualization, methodology, formal analysis and writing – review and editing. Oscar Rysavy and Patrick Barlow: methodology, formal analysis and writing – review and editing. Cláudio Rodrigues Leles: conceptualization, methodology, formal analysis and writing – review and editing. Túlio Eduardo Nogueira: conceptualization, methodology, formal analysis, investigation, resources, writing – review and editing, visualization, supervision and project administration.

## Funding

The authors have nothing to report.

## Conflicts of Interest

The authors declare no conflicts of interest.

## Supporting information


**Table S1:** Characteristics of the participating Dentistry undergraduate courses and participant distribution by Higher Education Institutions (HEI)* (*n* = 795).

## Data Availability

The data that support the findings of this study are available from the corresponding author upon reasonable request.
