# Supplementary material for: Ageism Against Older Adults and Associated Factors: A Multicenter Cross‐Sectional Study Among Dental Students in Midwestern Brazil
Source: Eur J Dent Educ. 2026 Apr 5;30(4):1740–50. doi: 10.1111/eje.70153 (PMC13686673; doi:10.1111/eje.70153)
Supplement: Supplementary file 1 [file EJE-30-1740-s001.docx]

**Tables**

| **Supplementary table 1.** Characteristics of the participating Dentistry undergraduate courses and participant distribution by Higher Education Institutions (HEI)* (n=795). | | | | |
| --- | --- | --- | --- | --- |
| **HEI** | **Type of HEI** | **Duration of the course** | **Academic component of geriatric dentistry** | **Study participants**  **n (%)** |
| 1 | Public | 5 years | Yes | 140 (17.6) |
| 2 | Private | 4 years | No | 100 (12.6) |
| 3 | Private | 5 years | No | 112 (14.1) |
| 4 | Private | 4 years | No | 160 (20.1) |
| 5 | Private | 4 years | No | 91 (11.4) |
| 6 | Private | 5 years | No | 173 (21.8) |
| 7 | Private | 5 years | No | 19 (2.4) |
| *Data extracted from each HEI website. | | | | |
